# Mycosis fungoides and Kaposi’s sarcoma association in an HIV-negative patient*

**DOI:** 10.1590/abd1806-4841.20164401

**Published:** 2016

**Authors:** Maria Carolina Prado Fleury Bariani, Luiz Fernando Fróes Fleury Júnior, Ana Maria Quinteiro Ribeiro, Siderley de Souza Carneiro, Tiago Arantes Pereira

**Affiliations:** 1 Universidade Federal de Goiás (UFG) – Goiânia (GO), Brazil

**Keywords:** Humanherpesvirus 8, Mycosis Fungoides, Neoplasms, Kaposi’s sarcoma

## Abstract

The association of mycosis fungoides and kaposi’s sarcoma in HIV-negative patients is a rare phenomenon. The presence of human herpesvirus 8 (HHV-8) – associated with all forms of Kaposi’s sarcoma – has also been recently identified in mycosis fungoides lesions. However, a causal association between HHV-8 and the onset of mycosis fungoides has not been established yet. The present case reports a patient who developed Kaposi’s sarcoma lesions after a two-year UVB phototherapy to treat a mycosis fungoides. Negative immunohistochemistry staining for Kaposi’s sarcoma-associated herpesvirus in the initial mycosis fungoides lesions strengthens the absence of a link between Kaposi’s sarcoma-associated herpesvirus and mycosis fungoides. Immunosuppression caused by the lymphoma and prolonged phototherapy were probably the contribut ing factors for the onset of Kaposi’s sarcoma.

## INTRODUCTION

Mycosis fungoides (MF) is the most common form of cutaneous T-cell lymphoma. It presents itself mostly in the early stages. The evolution to more severe forms is variable and its association with secondary malignancies may occur. Lymphoproliferative disorders are the most common diseases after the diagnosis of MF.^1,2,3^ Kaposi’s sarcoma (KS), in turn, is associated with human herpesvirus 8 (HHV-8) and is more common in transplanted patients, HIV patients, or patients on immunosuppressive treatments. The association of KS with lymphoproliferative disorders is unusual and less frequent in MF.^4^ The coexistence between MF and KS in HIV-negative patients is rare: the literature reports only a few records of this association.^5^ The influence of HHV-8 (present in all forms of KS) on MF occurrence has also been recently identified. However, there is no consensus about the role of the virus in the pathogenesis of MF and the development of secondary malignancies.^6,7,8,9^

## CASE REPORT

**Figure 1:**
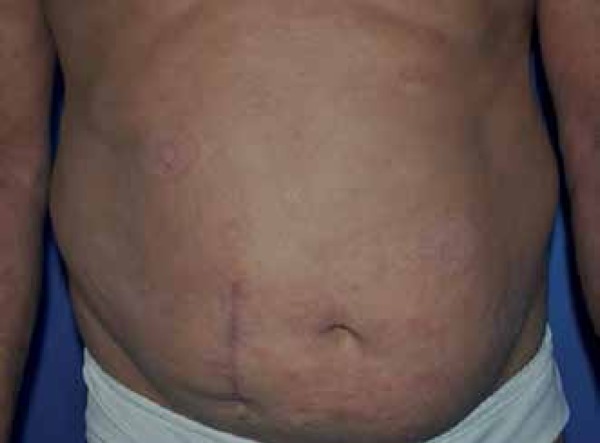
Mycosis fungoides – papules and erythematous, scaly plaques, not well defined, with slight infiltration in the abdomen

**Figure 2:**
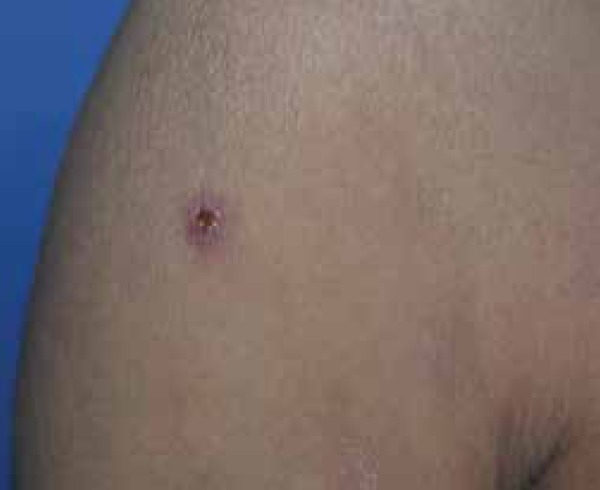
Kaposi’s sarcoma - well defined violaceous papules with central ulceration on the anterior side of the right arm

We report a 53-year-old male patient with a three-year history of mycosis fungoides stage IB. The patient was on his 138th narrowband UVB phototherapy session (two sessions per week) with partial control of the skin condition. Two years after the initial diagnosis, the patient developed fast-growing nodular lesions on the limbs. Dermatological examination revealed erythematous scaly plaques on the abdomen and thighs and violaceous papules on the anterior side of the right arm, lateral side of the left forearm and posterior side of the right leg (Figures 1 and 2).

**Figure 3:**
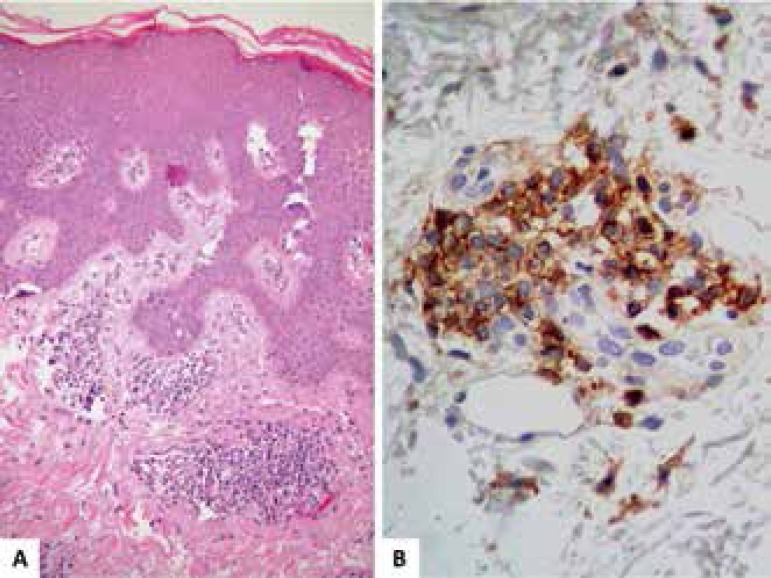
Mycosis fungoides - **A.** Moderate perivascular lymphocytic infiltrate with epidermotropism in a skin without spongiosis signals (HE - 100x). **B.** - Immunohistochemical marking for CD4 lymphocytes shows lymphocytes marking suggesting monotypic lymphoid infiltrate (400x)

**Figure 4:**
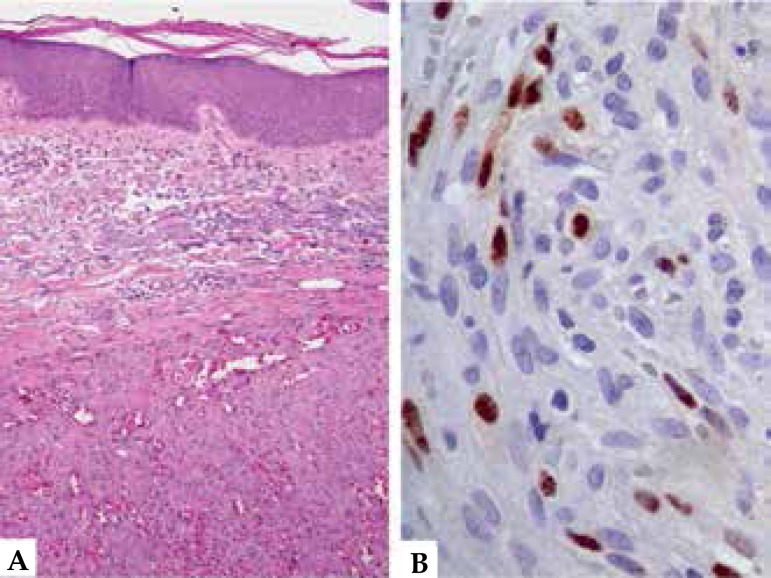
Kaposi’s sarcoma A. Well-defined vascular proliferation with expansive borders and vascular slits of different sizes, outlined by atypical endothelial cells (HE - 100x). B. Immunohistochemistry for HHV-8 shows marking in the endothelial cells (400x)

**Figure 5:**
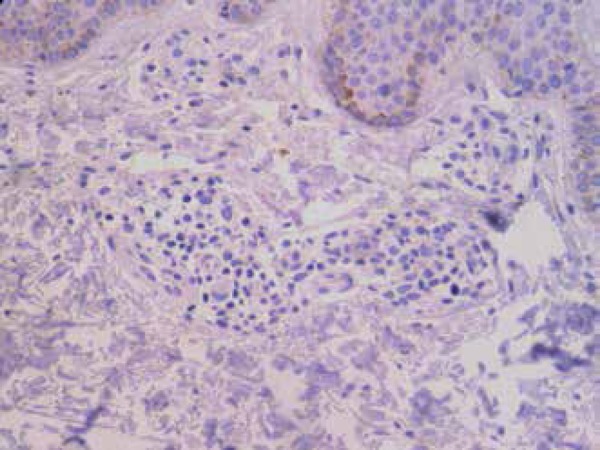
Immunohistochemistry in mycosis fungoides initial lesion showed no reactivity for HHV-8 in infiltrated lymphocytes (400x)

A biopsy revealed superficial perivascular dermatosis with some mildly pleomorphic lymphoid cells and positive immunohistochemistry for CD3, CD4 and CD5, negative for CD8, suggesting MF (Figure 3). Histology of violaceous papules showed a vascular lesion with fusiform cells and formation of vascular slits, compatible with KS. Immunohistochemistry also confirmed the diagnosis with positive HHV-8, CD31 and CD34 (Figure 4). Serology for HIV, HTLV and hepatitis was negative and staging tests were normal. The MF stage was maintained and KS was considered exclusively cutaneous. Immunohistochemistry was negative for HHV-8 in the MF material (Figure 5).

We opted for the surgical removal of the KS lesions and for the continuation of phototherapy for MF. During follow-up, new KS lesions occurred on the lower limbs, confirmed by biopsy and immunohistochemistry. We scheduled the excision of these new lesions. After three months, the lesions to be excised resolved spontaneously.

## DISCUSSION

Mycosis fungoides (MF) association with other malignancies is well established. MF patients present a 1-2 fold risk for developing secondary neoplasias.^1,3^ Hodgkin’s and non-Hodgkin’s lymphomas are the most common. Rarely, MF patients can develop cancer in the lung, colon, bladder, bile duct, vulva, skin (melanoma), and blood (acute myeloid leukemia).^1,2,3^ The coexistence of MF with KS – especially in negative-HIV and non-transplant patients – is rare. The reason for this association is not well established.^5^

MF, even at early stages, causes impaired cell-mediated immunity by T-cell activation. This factor could contribute to the development of other neoplasias.^2,3,4^ In the case of KS, that immunomodulation could favor the activation and replication of HHV-8, contributing to the emergence of clones of malignant cells and of KS lesions.

Genetic factors, environmental exposures and viral infections may also contribute to the occurrence of other neoplasias.^2,3,8^ A higher frequency of HLA-DRB1*11 was observed both in MF patients as in KS patients.^5^ Epstein Barr virus, cytomegalovirus and HTLV have also been identified in patients with MF. However, there is still controversy about the role of these viruses in the predisposition to secondary neoplasias.^8^

The recent detection of HHV-8 in patients with MF lesions suggests an influence of the virus in the pathogenesis of the disease and a likely predisposition to develop KS.^6,7,9^ HHV-8 could act as a chronic antigen or oncogene and induce the proliferation and uncontrolled activation of T-cells in the skin. Thus, it could contribute to the onset or development of MF.^6,7,8^ However, some authors believe that the presence of the virus is not necessarily related to the pathogenesis of the disease. The detection of the virus could only indicate the existence of opportunistic, recent, or reactivated infection.^8,9^ In the reported case, as observed in other studies, HHV-8 was not identified in the early MF lesions, strengthening the hypothesis that the virus presents no relation with the development of the disease.

Frequent exposure to treatments such as phototherapy, chemotherapy and radiotherapy could also contribute to increased risk of secondary neoplasias.^3,4,9^ Phototherapy induces local and systemic immunosuppression by DNA damage, Langerhans cell reduction and altered cellular immunity, with consequent generation of cytokines and T-cells with suppressing activities.^2,10^ In our case, the cumulative exposure to UVB radiation may have been a facilitating factor and immunosuppression inducer for the further development of KS.^10^

In the present case, we demonstrated the absence of association between HHV-8 and MF. Immunosuppression generated by MF, even at early stages of the disease, may predispose to the occurrence of other malignancies. Phototherapy may aggravate this immunosuppression when held for prolonged periods. Physicians should be alert to any changes of cutaneous or systemic clinical pictures of their patients with MF for early detection of other neoplasias.
